# Automatic Segmentation of Laser-Induced Injury OCT Images Based on a Deep Neural Network Model

**DOI:** 10.3390/ijms231911079

**Published:** 2022-09-21

**Authors:** Tianxin Gao, Shuai Liu, Enze Gao, Ancong Wang, Xiaoying Tang, Yingwei Fan

**Affiliations:** 1 School of Life Science, Beijing Institute of Technology, Beijing 100081, China; 2 School of Medical Technology, Beijing Institute of Technology, Beijing 100081, China

**Keywords:** optical coherence tomography, deep neural network, image segmentation, laser-induced skin injury

## Abstract

Optical coherence tomography (OCT) has considerable application potential in noninvasive diagnosis and disease monitoring. Skin diseases, such as basal cell carcinoma (BCC), are destructive; hence, quantitative segmentation of the skin is very important for early diagnosis and treatment. Deep neural networks have been widely used in the boundary recognition and segmentation of diseased areas in medical images. Research on OCT skin segmentation and laser-induced skin damage segmentation based on deep neural networks is still in its infancy. Here, a segmentation and quantitative analysis pipeline of laser skin injury and skin stratification based on a deep neural network model is proposed. Based on the stratification of mouse skins, a laser injury model of mouse skins induced by lasers was constructed, and the multilayer structure and injury areas were accurately segmented by using a deep neural network method. First, the intact area of mouse skin and the damaged areas of different laser radiation doses are collected by the OCT system, and then the labels are manually labeled by experienced histologists. A variety of deep neural network models are used to realize the segmentation of skin layers and damaged areas on the skin dataset. In particular, the U-Net model based on a dual attention mechanism is used to realize the segmentation of the laser-damage structure, and the results are compared and analyzed. The segmentation results showed that the Dice coefficient of the mouse dermis layer and injury area reached more than 0.90, and the Dice coefficient of the fat layer and muscle layer reached more than 0.80. In the evaluation results, the average surface distance (ASSD) and Hausdorff distance (HD) indicated that the segmentation results are excellent, with a high overlap rate with the manually labeled area and a short edge distance. The results of this study have important application value for the quantitative analysis of laser-induced skin injury and the exploration of laser biological effects and have potential application value for the early noninvasive detection of diseases and the monitoring of postoperative recovery in the future.

## 1. Introduction

Optical coherence tomography (OCT) is a noninvasive imaging method that can obtain high-resolution three-dimensional volume images of biological tissues [1]. The principle of OCT is similar to ultrasonic imaging, but the difference is that it uses light rather than sound waves. Image acquisition is based on light with a short coherence length, and the distance of the partial reflection structure in biological tissue is measured by an interferometer. OCT is a rapidly developing imaging technology that is used in various biological and medical disciplines. Especially in ophthalmology, OCT has been used as an imaging method for diagnosing retinal diseases since the early 1990s [2]. Later, OCT was introduced into dermatology as a noninvasive option for biopsy and histology. In addition to routine application in the clinic, OCT can also be used for the study of animal models in vivo rather than human bodies.

OCT can perform noninvasive, high-resolution, two-dimensional, or three-dimensional cross-sectional imaging of the microstructure of biological tissue in situ. Conventional OCT with a horizontal resolution of 10–15 μm can usually identify the cuticle, epidermis, and upper dermis of hairless skin (palms and soles), as well as skin accessories and blood vessels [3]. For example, OCT can be used to noninvasively monitor skin inflammation, hyperkeratosis, and light adaptation. Polarization-sensitive OCT (PS-OCT) [4,5,6,7,8] can detect layered and well-organized collagen in normal skin and collagen fiber tissue disorders in the dermis, which can be caused by pathology such as burns and basal cell carcinoma (BCC). OCT is an effective imaging method for the diagnosis of keratinocyte carcinoma (nonmelanoma skin cancer), which has the potential for noninvasive detection of early diseases [9]. OCT has been widely used to diagnose BCC, which is the most common form of malignancy in humans [10]. A potential application of OCT is to determine the tumor margin of basal cell carcinoma before surgery, which can reduce the number of stages required for staged tumor resection [11,12,13,14]. OCT may also play a role in monitoring the recurrence of basal cell carcinoma after local treatment [15,16], photodynamic therapy [17,18], and laser treatment [19], all of which are related to a much higher recurrence rate than surgical resection. OCT has also been used to evaluate the response of basal cell carcinoma to systemic hedgehog inhibitors [20]. Squamous cell carcinoma (SCC) of the skin is another common keratinizing cell carcinoma. In contrast to basal cell carcinoma, SCC has metastatic potential. Early identification and treatment are essential to reduce this risk. The application of OCT in the diagnosis of SCC and the differentiation of preinvasive diseases (actinic keratosis (AK) and Bowen’s disease) and invasive SCC35 has been evaluated [21,22,23,24]. The morphological features of squamous cell carcinoma and preinvasive diseases include thickening or disorganization of the upper epidermis, destruction of the normal skin layer, and abnormalities at the dermal epidermal junction [15].

OCT is a noninvasive method to monitor skin damage in the clinical environment. By using ultrahigh-resolution optical coherence tomography [3], skin wound biopsy was performed on the back surfaces of seven mice with a full thickness perforator at a diameter of 2.5 mm. OCT imaging was performed to evaluate structural features related to the healing process. The OCT results were compared with the corresponding histology. Two automatic quantitative analysis methods were used to recognize the dermal epidermal junction and segment OCT images. The characteristics of skin wound healing, such as wound size, epidermal migration, the formation of dermal epidermal junctions, and the difference in wound composition, are easy to recognize in OCT images. Blister formation was also observed. Preliminary results showed that OCT is a feasible tool for noninvasive monitoring of wound healing in vivo. Lu J. et al. analyzed the optical attenuation coefficient of OCT images of burned skin [25] to strengthen the contrast of scanned images and to promote the automatic segmentation of the epidermis from the scanned images and serve as an objective index for the evaluation of burn healing. Ahn Y et al. proposed a quantitative tissue monitoring method based on OCT [26] to evaluate tissue regeneration after laser radiation. However, the tissue regeneration was evaluated only according to the color morphology in the observed image, and the damaged part was not segmented. Line field confocal optical coherence tomography (LC-OCT) imaging can detect pustular structures with high accuracy, including shape, edge, morphology, and cell content, as well as unique epidermal and accessory changes in different cases [27]. Kavita Dubey et al. detected human skin thermal injury based on a classifier by separating the morphological features extracted from polarization-sensitive optical coherence tomography (PS-OCT) images and achieved good results [28].

Fischman et al. trained a deep learning algorithm to segment keratinocyte (KC) nuclei from line field confocal optical coherence tomography (LC-OCT) 3-D images [29]. Based on these fragments, a series of quantitative, repeatable, and biologically related indicators were derived to describe KC nuclei separately. Ji Y et al. used a deep learning framework to automatically detect the epidermis and scab layer and verified the effectiveness of the deep learning method by comparing the segmentation results obtained by the algorithm with the gold standard method (manual segmentation). The proposed deep learning method showed good results in segmentation accuracy and automatic quantification of epidermal and scab thickness of mouse skin data within the standard healing timeline, expanding the role of OCT in clinical and research fields [30]. Using the method of deep learning [31], the segmentation of hair follicle skin gland units from epidermal OCT images of healthy humans is of great significance in the progression of skin diseases such as folliculitis, acne, lupus erythematosus, and basal cell carcinoma. A system based on OCT and depth learning was proposed to automatically measure the internal volume of hydrogels [32]. When deep learning did not occur, the skin layer segmentation of skin OCT images and image enhancement based on morphology, such as gradient information, was utilized to monitor the edge of the skin layer [33]. Kepp T. et al. used a deep convolutional neural network to realize the automatic segmentation of three skin layers in mouse skin OCT image data [34], but this research did not explore the field of laser skin injury. Chou H.Y. et al. used a convolutional neural network to monitor the skin epidermal junction (DEJ) [35]. At present, there are few studies on the intelligent segmentation and recognition of OCT images of laser skin damage and repair.

In this study, the segmentation and quantitative pipeline of laser-induced skin damage OCT images based on a deep learning framework were carried out, and the hierarchical labels of skin OCT images were obtained through manual annotation. By using the U-Net method, the original OCT image is used as the input to realize the accurate segmentation of each layer of mouse skin, and the pixel prediction of each class of label was output. The noninvasive detection of skin by using the OCT system and the accurate segmentation of skin OCT images by a deep learning network are of great significance for the noninvasive detection of early diseases, provide effective guidance for the surgical treatment of skin diseases, and have potential value for noninvasive monitoring after surgery.

## 2. Result

### 2.1. Results of OCT Image Preprocessing

**Figure 1:**
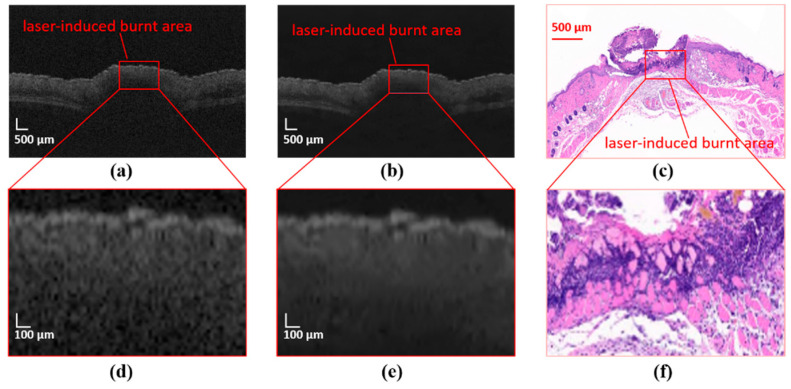
OCT image and label. (**a**) Original OCT image and (**d**) corresponding enlarged ROI. (**b**) Preprocessed OCT images and (**e**) corresponding enlarged ROI. (**c**) Histological sections and (**f**) corresponding enlarged ROI.

OCT images usually have defects such as low contrast and serious noise pollution. As shown in Figure 1a, the original image is a grayscale image, and the boundary between each skin layer is not obvious and has speckle noise. To enhance the difference of each region that needs to be segmented in the OCT image of mouse skin, preprocessing is carried out before the image is input into the deep learning network model. The preprocessed OCT image is shown in Figure 1b. The noise is reduced, the whole image becomes clearer, and the stratification is more obvious. The boundary of the damage area is also clearer.

### 2.2. Analysis Results of OCT Image Segmentation of Mouse Skin Injury

**Figure 2:**
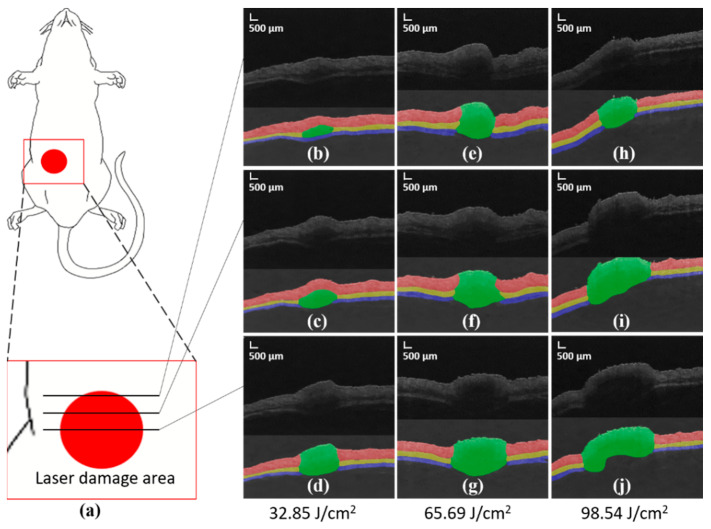
(**a**) It is the model diagram of the injured part of the mouse. (**b**–**j**) Segmentation results corresponding to radiation doses of 32.85 J/cm^2^, 65.69 J/cm^2^, and 98.54 J/cm^2^, respectively.

According to the model structure and the labels corresponding to the data in the training set, the network is trained. Finally, the test set is input into the network to obtain the prediction segmentation results. The original OCT image of mouse skin, the segmentation results of manual marking, and the segmentation results predicted by the neural network model used in this study are compared. The experimental results are shown in Figure 2. In the prediction results, we can clearly see the stratification of mouse skin and the thickness distribution of each layer of skin.

Figure 2b–d,e–g,h–j shows the segmentation results of OCT images damaged by laser radiation duration of 1, 2, and 3 s, respectively. Each image contains two parts, the upper part is the B-Scan image, and the lower part is the segmentation result.Each column of image is from the edge of the damage to the center of the damage from top to bottom. Figure 2b–d shows that the damage caused by the 1 s radiation dose has the smallest damage area and the shallowest depth. The upper dermis at the edge of the injury was not significantly damaged, but the fat layer below the dermis was damaged. In the central area of the injury area, the dermis, fat layer, and muscle layer were significantly damaged. Figure 2e–g shows that the damage area is larger than the one-second radiation dose. The dermis began to be damaged at the edge of the injury areas, and the muscle layer at the lowest layer was also damaged and punctured by laser. Figure 2h–j shows that the damage span of the 3 s radiation dose increased significantly compared with that of the 2 s radiation dose. Given the depth limitation of the OCT imaging system, this can only be judged according to the fracture of the muscle layer, and the laser radiation completely damages the muscle layer. With an increase in the radiation dose, the damaged area expanded and deepened.

**Figure 3:**
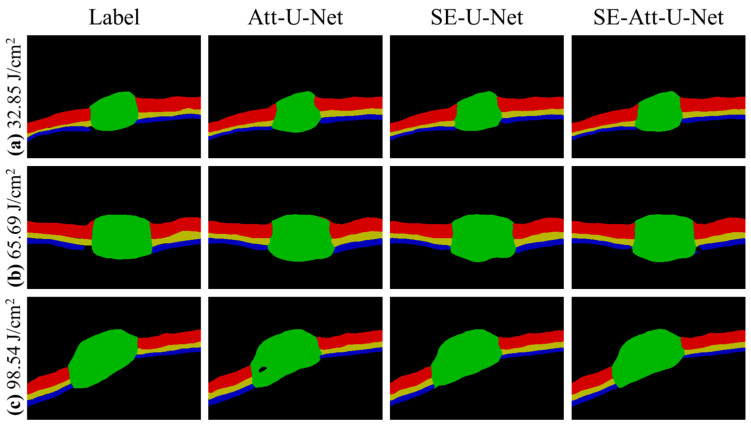
(**a**–**c**) Segmentation results corresponding to radiation doses of 32.85 J/cm^2^, 65.69 J/cm^2^, and 98.54 J/cm^2^, respectively.

To further enhance the segmentation effect and highlight the details of the damaged edge, the attention mechanism module, SE attention mechanism module, and U-Net structure are combined to enhance the segmentation. A comparison of results from different network models is shown in Figure 3. Figure 3a–c shows the segmentation results of OCT images damaged by radiation doses of 32.85 J/cm^2^, 65.69 J/cm^2^, and 98.54 J/cm^2^ with different neural network models. The training time of 50 epochs is about 40 min for each model. U-net is a little faster than the other methods with more modules.

### 2.3. Evaluation Results of Different Segmentation Network Models

The OCT prediction segmentation map of mouse skin obtained by the neural network model is evaluated. The manually labeled label map is used as the evaluation standard, and the red, yellow, blue, and green areas of each OCT image are evaluated and calculated separately. The basic situation of each evaluation parameter is shown in Table 1. The Dice coefficient represents the overlap rate between the prediction and truth labels. From Table 1, each network has the best effect on dermis segmentation, among which the Att-SE-U-Net model has the best effect, and its Dice coefficient reaches 0.93. From the perspective of the overlap rate, the original U-Net network has the highest segmentation effect on the damaged area, and its Dice coefficient reaches 0.92. The ASSD describing the edge distance of each area is fewer than five pixels in the three skin layers. In the ASSD evaluation of the damaged area, the network model with the addition of attention and the SE attention mechanism module has been greatly improved, and the distance of two pixels is reduced compared with the basic network framework of the U-Net model. In the HD evaluation that describes the maximum edge distance, the network advantage of adding the attention mechanism modules is more obvious and is mainly reflected in the improvement of the segmentation effect of the damaged areas.

**Figure 4:**
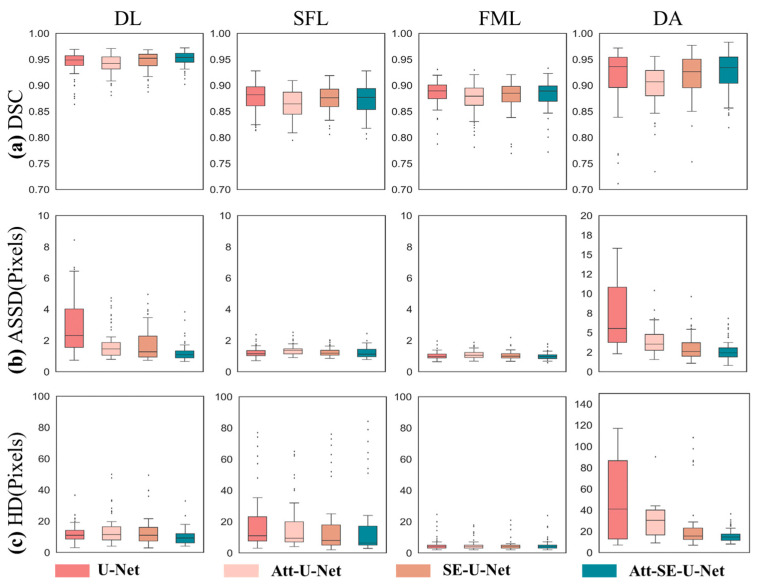
Box diagram of quantitative evaluation results of deep convolutional network models. (**a**) DSC, (**b**) ASSD, and (**c**) HD.

To better analyze and observe the segmentation effect of each network model on different regions, the results of the validation set segmented by all network models are sorted into a box diagram for analysis, as shown in Figure 4. It can be seen from the Figure that the increase in the attention mechanism module has significantly improved the segmentation of damaged areas. In the evaluation of the Dice coefficient, the network model with the added attention mechanism module did not significantly improve or decrease the segmentation effect of each area, but it can be seen from the box diagram that the segmentation effect of the models with the added attention mechanism is more concentrated and stable. In the assessment indicators of ASSD, it can be seen from the box diagram (Figure 4) that the segmentation effect of the attention mechanism module on the dermis and the injured area is the most obvious, and the method of combining the two attention mechanism modules made in this study has the most obvious effect. In the HD evaluation index, it can be seen from the box diagram that the segmentation effect of the attention mechanism module on the three skin layers improved only slightly, and the segmentation effect of the damaged area improved significantly, reducing its distance from 100 pixels to no more than 20 pixels.

### 2.4. Three-Dimensional Visualization

**Figure 5:**
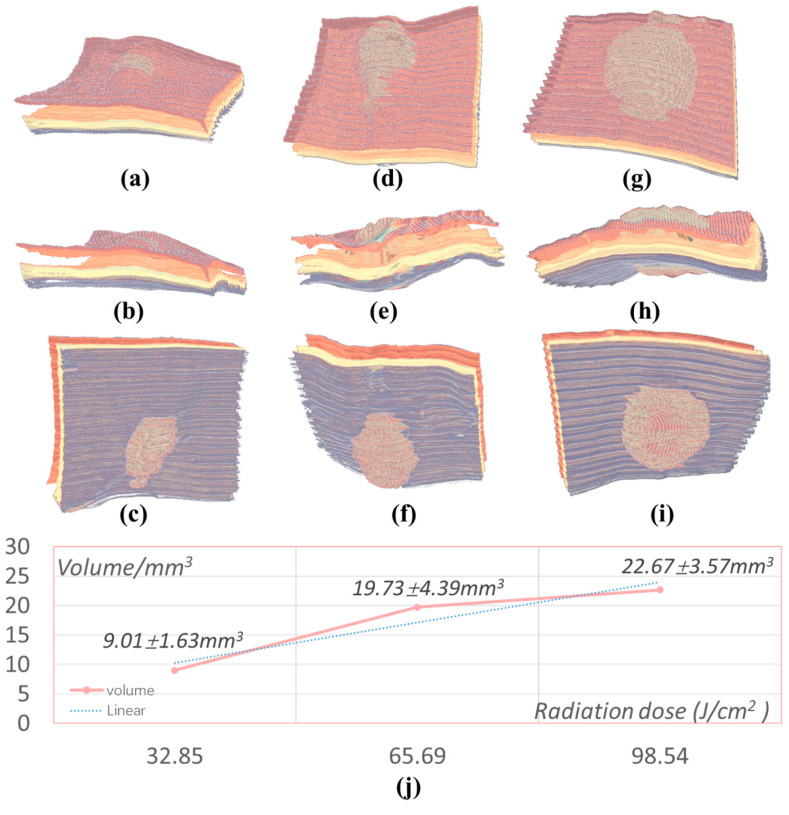
Three-dimensional visualization and quantitative analysis. (**a**–**i**) Different views of 3-D reconstruction rendering and visualization and (**j**) areas of burned skin (in mm^3^).

Using the above trained network model, the OCT images of the skin tissues and damaged areas induced by radiation doses of 32.85 J/cm^2^, 65.69 J/cm^2^, and 98.54 J/cm^2^ are segmented and used to conduct a 3-D reconstruction of the segmented fault sequence. The reconstruction results are shown in Figure 5. In terms of morphological structure, Figure 5a–c shows the reconstruction results of the damaged area with a radiation dose of 32.85 J/cm^2^, and the perspective corresponds to observing the skin structure from three different angles: dermis, fat layer, and muscle layer. By adjusting the transparency, the edge information of the damaged area in the skin layer can be observed clearly.

Figure 5d–f shows 3-D reconstruction images of the damaged area of two radiation doses. Figure 5g–i shows the images of three angles of three-dimensional reconstruction of the damaged area of three radiation doses. It can be clearly seen from the 3-D reconstruction images of the damaged skin. With an increase in the number of radiation doses, the volume of the damaged areas also increases. Figure 5j shows statistical results of the volume of the injury area at three different doses. The volume of the injury area corresponding to different radiation doses was 9.01 ± 1.63 mm^3^, 19.73 ± 4.39 mm^3^, and 22.67 ± 3.57 mm^3^, respectively. Hence, the quantitative analysis of laser-induced skin damage is constructed through segmentation using deep convolutional networks and volume calculation using 3-D visualization. A quantitative intelligent evaluation method is proposed to study laser-induced damage and laser biological effects. This method can be used to assist in the screening of skin diseases and skin damage.

## 3. Discussion

In this study, a deep learning network architecture based on U-Net and its improved model was used to achieve accurate segmentation of OCT images of laser-irradiated mouse skin, including the dermal epidermal layer, subcutaneous fat layer, muscle layer, and damaged area. A pipeline of segmentation and quantitative analysis of laser-induced OCT images was proposed using a deep neural network. The Dice coefficient, ASSD, and HD were used to analyze the segmentation results. According to the evaluation results, the Att-SE-U-Net model has the best segmentation performance. Three-dimensional reconstruction is further used to quantitatively analyze the size and volume of damaged areas under different laser radiation doses and obtain the quantitative results.

In this study, the intelligent segmentation of OCT images of mouse skin after laser injury was achieved for the first time as far as we know, and the area of skin injury and the layered structure of mouse skin under different laser radiation doses was studied. Compared with our previous study [36], this study does not use the optical attenuation coefficient of OCT images to analyze the skin and its damaged structure, and the damaged area and the layered tissues of different skin can be directly obtained without analyzing the optical attenuation of the damaged structure. Therefore, this study has the following advantages. First, the analysis of the optical characteristics of the tissue is exempted, and the layered structure and damaged area of the skin are obtained directly according to the experience of the pathologist. Second, this study realizes intelligent and automatic stratification and damage area rendering and obtains the damage area boundary information. Finally, this study achieves a quantitative evaluation of several models through relevant quantitative evaluation methods, obtains a performance comparison of segmentation of the skin layers and damaged area segmentation, further obtains the optimal model under the current type of data, and proves the reliability of this dual attention mechanism-based U-Net model.

In terms of imaging evaluation technology of different laser injury doses, this study proposed an intelligent segmentation algorithm for OCT images of laser injury based on a neural network model. U-Net and its improved model were used to achieve segmentation, which further realized the quantitative evaluation of several models. Previous studies [32] achieved only intelligent segmentation of OCT images of different skin layers based on the U-Net deep neural network model, while this study achieved accurate segmentation of damaged mouse skin structures under different doses of laser radiation and different skin layers. In other words, this study proposed an imaging evaluation method based on a deep neural network model for the biological effects of laser radiation damage. This method is significantly different from the evaluation method of pathology [37,38]. In the future, the effect of high-resolution imaging can replace the pathological evaluation method to achieve in vivo, noninvasive, and rapid real-time evaluation.

According to different network models, the results of this study represent an important breakthrough in the field of treating laser skin damage. The performance of the network models based on the attention and SE mechanism modules improved in terms of segmentation; among them, the DSC reached 0.93 in the division of DL, and all showed a higher level than the other three network models. This method can improve the accuracy of intelligent segmentation. Therefore, this study for the first time realizes the development of an intelligent segmentation algorithm using network models for high DSC values and a double attention (SE and attention) mechanism module, constructs an effective intelligent segmentation model for laser skin injury, and provides an intelligent segmentation method and quantitative evaluation means for the future study of laser biological effects. Furthermore, the intelligent segmentation algorithm based on deep neural network models provides an effective diagnostic method for skin lesion identification and quantitative evaluation of treatment [18]. The results of this study lay a theoretical foundation for the intelligent diagnosis of OCT images and have important application potential in the structural recognition of skin OCT images. In the future, the method of this study can provide an effective analysis scheme for the recognition of structural lesions in layered skin OCT images.

**Figure 6:**
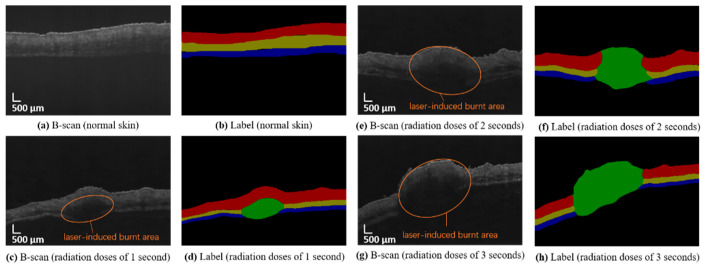
OCT image of mouse skin and manual labeling. (**a**,**c**,**e**,**g**): OCT images immediately after radiation corresponding to radiation doses of 1, 2, and 3 s, respectively. (**b**,**d**,**f**,**h**): labels corresponding to images of (**a**,**c**,**e**,**g**).

As shown in Figure 6c,d, damage occurs in the deep region with intact surface. The laser beam is collimated. The diameter of the laser spot was approximately 1.5 mm. This may be because of the different tolerance thresholds to injury of different tissues. The fat layer is more easily damaged than the dermis by the heat caused by irradiation. Cross validation can optimize parameters and prevent model heterogeneity caused by data set division. However, considering that n-fold cross validation is adopted, although model parameters may change, the model itself has not changed substantially. Data in practical applications are not limited to existing data. Even if cross validation is used, it may not necessarily give optimization parameters a priori. So, we do not use cross validation.

In this study, OCT images of laser-damaged mouse skin were discussed and quantitatively analyzed based on a deep convolutional network. However, the limitations are as follows: the amount of data is small, the model does not have enough samples to distinguish features, and data overfitting is prone to occur, resulting in low training errors but high-test errors. The quality of the dataset needs to be improved. Due to the limitation of OCT imaging resolution (longitudinal resolution is approximately 12 μm), ultrahigh accuracy has not been achieved thus far, and there are problems with the correct identification of interference models. Due to the limitations of the depth of OCT imaging, the specific depth of laser damage was not well-analyzed. Here, the depth of damage was mainly determined according to the fracture of each skin layer. When calculating the volume of the damaged area in mice, due to the difference in skin layer thickness in different mice, the volume of the damaged area in different mice under the same radiation dose was greatly different. This is not conducive to the quantitative analysis of the damaged area in mice. In the future, the algorithm of image processing and analysis will be improved in order to increase the segmentation accuracy. An intelligent segmentation algorithm specifically for skin OCT images will be proposed to achieve accurate segmentation of multilevel and damaged areas. Furthermore, a 3-D reconstruction algorithm will be used to visualize the experimental data and assist real-time intraoperative imaging. It is hard to measure injury volume in vivo. We have not confirmed the injury volume using other equipment than OCT. Further study may be carried out to verify the method.

## 4. Methods and Materials

In this study, a sweep source OCT (SS-OCT) imaging system was used to image the abdominal skin of mice, and a U-Net network combined with a variety of attention mechanism modules was used to realize the multicategory segmentation of mouse skin OCT. The OCT images of dermis, subcutaneous fat layer, muscle layer, and laser-damaged parts of mouse skin were segmented layer-by-layer, and the average surface distance (ASSD) was calculated. The Hausdorff distance (HD) and Dice coefficient were used to evaluate the performance of these deep network models. Finally, the three-dimensional OCT images of the scanned area were reconstructed, and the volume of the damaged area was calculated to obtain the layered structure of the skin and the range and volume information of the damaged area.

### 4.1. Experimental Setup and System Configuration

The weight of 4- to 5-week-old adult BALB/c-mu mice was approximately 15 g. The experimental setup and system configuration were the same as those in our previous research [36]. The abdominal skin of mice was radiated with a supercontinuum laser, and damaged areas with different degrees of injury were generated by controlling the laser radiation dose. In this experiment, three different doses of radiation were carried out corresponding to 1, 2, and 3 s radiation times, and then OCT images of the subcutaneous nondamaged areas and damaged areas of mice were collected. The Beijing Institute of Radiation Medicine Experiment Animal Center-Approved Animal Protocols approved this study. All animal experiments were performed in accordance with the guidelines in IACUC-DWZX-2019-502.

**Figure 7:**
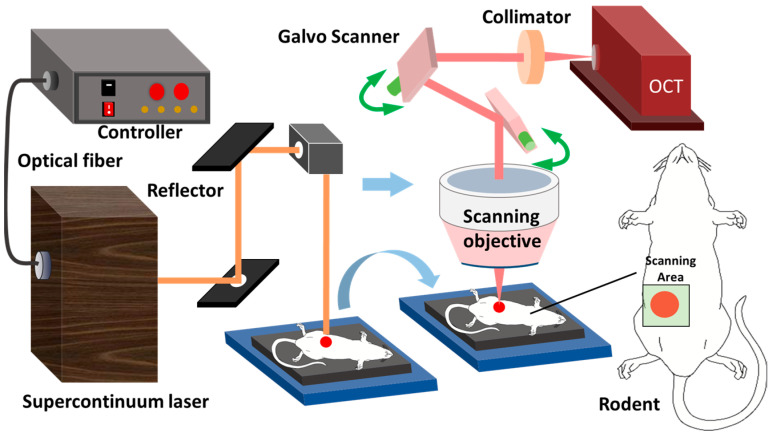
System setup: optical path diagram of laser radiation on skin of living mice and SS-OCT imaging system for acquiring OCT images.

The output laser power (W) is P, and the radiation time (s) is T. The laser spot radius is D. We use the equations Q = PT and $H=\frac{4Q}{\pi D^{2}}$ to calculate energy Q and radiation dose H, respectively. Figure 7 shows the OCT scanning model and laser radiation light path on the skin of living mice. A power meter was used to measure the laser power. The radiation time was controlled by the shutter, which can control the radiation time through its opening and closing operations. The OCT module was used to image normal and laser-damaged skin and to establish thermal damage monitoring in vivo on animal skin. Mouse skin was irradiated with a supercontinuum laser (SuperK EXTREME series; NKTPhotonics in Denmark). The spectral width ranged from 400 to 2400 nm, in which case the output laser power was set to 0.445 W. The radiation time was controlled by the shutter and set to 1, 2, and 3 s. Using the above method, the laser radiation dose was set to three levels with a minimum output dose of 32.85 J/cm^2^ and maximum output dose of 98.54 J/cm^2^. Table 2 lists the radiation doses corresponding to radiation times of 1, 2, and 3 s (Table 2).

The scanning proportion of the home-built OCT module is sufficient to scan the field of view (FOV) of an approximately 10 mm × 10 mm damage point. Scanning source OCT (SS-OCT) is composed of a micro-electro-mechanical system (MEMS)-based scanning source (HSL-20-100-B, center wavelength: 1310 nm; Santec Technologies, AI Chin, Japan), a balanced photodetector (INT-MSI-1300B; Thorlabs, Newton, NJ, USA), data acquisition equipment (ATS9350; Alarztec Technologies, Newton, NJ, USA), and other components. The axial resolution of the image provided by OCT imaging in air is approximately 12 μm, and the horizontal resolution is approximately 22 μm. The image acquisition rate is approximately 60 frames per second.

### 4.2. OCT Imaging of Skin in Injured Area

The collected images are manually marked by experts according to the skin structure. There are more than 1000 B-scan OCT images used in this experiment, including more than 700 B-scan OCT images manually labeled for training. The OCT images and labels of mouse skin are shown in Figure 6. There are five categories: dermis layer (DL), subcutaneous fat layer (SFL), muscle layer (FML), damaged areas (DA), and background. These correspond to the colors red, yellow, blue, green, and black in the label.

### 4.3. OCT Image Segmentation Based on U-Net and Its Improved Network

**Figure 8:**
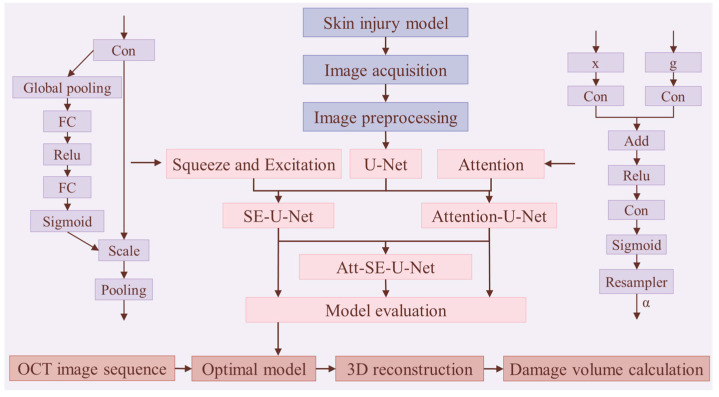
Flowchart of multicategory segmentation method for mouse skin OCT images.

In this study, the abdominal skin tissue of adult mice was collected, and OCT images were obtained. The OCT images of laser-induced damage were preprocessed, including the removal of background noise and speckle noise, to obtain high-quality OCT images of mouse skin. After preprocessing, the U-Net-based neural networks, which included the attention, SE attention, and dual attention mechanism modules, were used to segment OCT images of mouse rodent skin and damaged areas induced by laser radiation, and the segmentation performance of these network models was evaluated quantitatively and qualitatively to evaluate the networks’ performance. The process of the multicategory segmentation method based on the deep convolutional neural network model of mouse skin OCT images is shown in Figure 8.

#### 4.3.1. Mouse Skin OCT Image Preprocessing

The obtained original OCT image includes background and speckle noises. Here, a probability-based nonlocal mean filter [39] is used to remove speckle and background noises, and the bright stripes at the top of the image and the parts without information at the bottom of the image are cut to reduce noise interference and reduce the amount of calculation. Thus, high-precision and high-quality OCT images of mouse skin after laser injury can be obtained, from which the structure of different layers of skin and the structure of damaged areas can be clearly seen.

#### 4.3.2. Multiclassification Segmentation of Skin OCT Images Based on Deep Learning Network

This study uses a U-Net network model combined with the attention [40] and the squeeze-and-excitation [41] attention module to segment mouse skin OCT images into multiple categories. Four networks are used to segment the OCT images: U-Net, Att-U-Net, SE-U-Net, and dual attention mechanism-based U-Net (Att-SE-U-Net) models.

**Figure 9:**
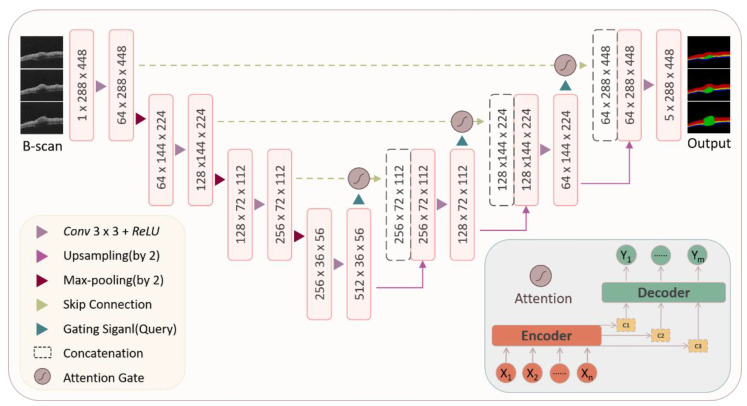
Network structure of attention-based U-Net (Att-U-Net) model.

The structure of the attention U-Net model is shown in Figure 9. The left half of the model is the encoder structure, which is composed of a convolution layer and pooling layer and is used to extract the features of different dimensions of the mouse skin OCT image. The right half of the model is the decoder, which is composed of upsampling and a convolution layer. The high-level semantic feature map obtained from the mouse skin OCT image is restored to the original resolution mouse skin OCT image. Furthermore, the skip connection structure is adopted, combined with the feature map obtained in the encoder and the feature map obtained in the decoder, in which the attention mechanism module is inserted. The more abstract OCT image features extracted through multiple convolution layers are combined with the features with higher resolution according to the parameters of the attention module, and finally, the segmentation results are output. The process of inserting the attention module during skip connection is as follows [Equation (1)]:

(1) $$W_{c}^{l}=\left(W_{i}^{l}\cdot \alpha_{l}\right):W_{u}^{l}$$

where $W_{c}^{l}$ represents the result of the skip connection, which is the result of the combination of the characteristic map and the upper sampling result, $W_{i}^{l}$ represents the lower sampling result, $W_{u}^{l}$ represents the upsampling result, and $\alpha_{l}$ represents the corresponding attention coefficient calculated by using the method mentioned in [35]. *l* represents the number of skip connection operations.

**Figure 10:**
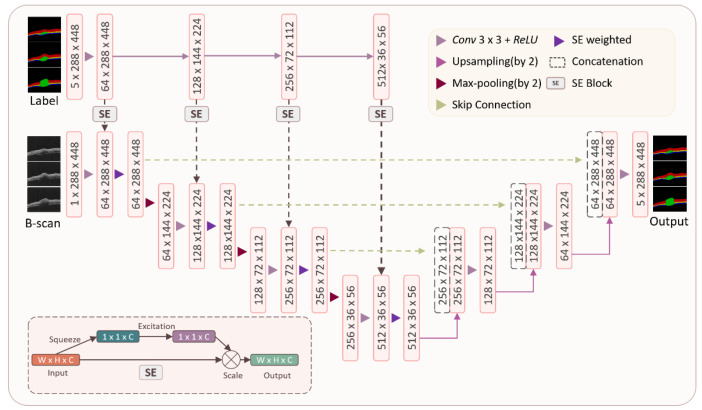
Network structure of squeeze-and-excitation-based U-Net (SE-U-Net) model.

The structure of the SE-U-Net model is shown in Figure 10. The left half of the model is an encoder structure composed of a convolution layer, pooling layer, and sequence and exception module. Based on the U-Net model, this structure adds an SE module, multiplies the parameters obtained by the SE module after convolution, and then performs a pooling operation. This is used to extract the characteristics of mouse skin OCT images corresponding to different channel information of labels. The right half of the model is a decoder, which is composed of upsampling and a convolution layer. The high-level semantic feature map obtained from the mouse skin OCT image is restored to the original resolution mouse skin OCT image. At the same time, the skip connection structure is used and combined with the feature map obtained in the encoder and the feature map obtained in the decoder. The more abstract OCT image features are extracted through multiple convolution layers and combined with higher resolution features. Finally, the segmentation results are output.

**Figure 11:**
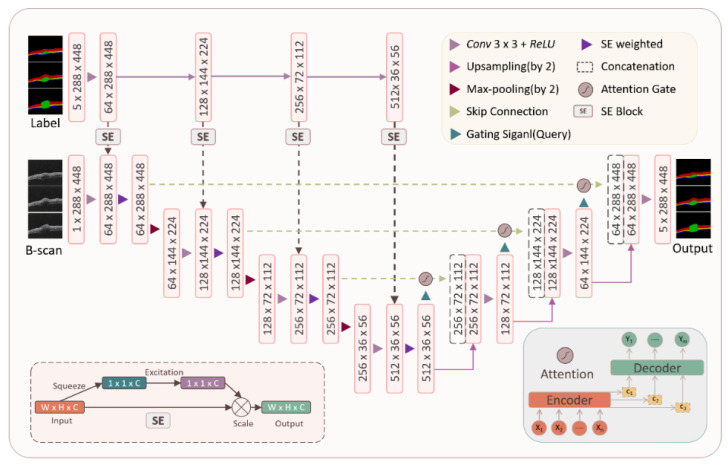
Network structure of dual-attention-mechanism-based U-Net model (Att-SE-U-Net).

We further propose an intelligent segmentation method of laser skin injury based on the Att-SE-U-Net model (Figure 11). The left half of the model is an encoder structure composed of a convolution layer, pooling layer, and sequence and exception module. Based on the U-Net model, this structure adds an SE module, multiplies the parameters obtained by the SE module after convolution, and then performs a pooling operation, which is used to extract the characteristics of mouse skin OCT images corresponding to different channel information of labels. The right half of the model is a decoder, which is composed of upsampling and a convolution layer. The high-level semantic feature map obtained from mouse skin OCT images is restored to the original-resolution mouse skin OCT images.

Furthermore, a skip connection structure is adopted and combined with the feature map obtained in the encoder and the feature map obtained in the decoder, in which the attention mechanism is inserted. The more abstract OCT image features of the skins extracted through multiple convolution layers are combined with the features with higher resolution according to the parameters of the attention module. Finally, the segmentation results are output. The model parameters are adjusted so that the model has the advantages of attention and SE modules applicable to the OCT images of damaged skin in this experiment and better achieves the accurate segmentation of OCT images.

This experiment is carried out on the Windows 10 system. This method was processed on a workstation with an Intel(R)Xeon(R)(R) Gold CPU (2.90 GHz) and two graphics cards. The graphics card is an NVIDIA Quadro RTX 5000. The model is coded in Python 3.8. The network model and the attention and SE mechanism module network models are constructed using the modules in PyTorch 1.8.0. The Dice loss and cross-entropy loss functions are used for comparison. The initial value of the model learning rate is 0.0001, and then it is reduced by an index of 0.9 according to the number of iterations.

#### 4.3.3. Quantitative and Qualitative Evaluation of OCT Image Segmentation

OCT image evaluation based on the U-Net network quantifies the segmentation results. First, the confusion matrix is calculated, and the segmentation results are evaluated using the *ASSD*, *HD*, and Dice coefficients. Then, the specific calculation method of each evaluation parameter is as follows.

The Dice similarity coefficient (DSC) is used to quantify the overlap between prediction and truth labels. The closer the value is to 1, the better the prediction effect of the network model. The calculation method is as follows [Equation (2)]:

(2) $$Dice=\frac{2\times \left(pred\;\cap \;true\right)}{pred\;\cup \;true}$$

where *pred *is the set of predicted values, and *true *is the set of real values.

The DSC calculation considers only the intersection of two groups of points and does not consider the distance between outer points. In addition, the size of the segmented region has an impact on DSC because misclassification has a greater impact on smaller regions than on larger regions. Therefore, the average symmetrical surface distance (*ASSD*) and Hausdorff distance (*HD*) are additionally used for evaluation. Let *S_P_* = {*p*_0_, …, *p*_n1_} and *S_G_* = {*q*_0_, …, *q*_n2_} be subsets of prediction segmentation P and basic truth value G, *S_P_* ⊆ *P*, and *S_G_* ⊆ *G*.

The surface distance *SD* between the *S_P_* and *S_G_* is defined as [Equation (3)]:

(3) $$SD\left(S_{P},S_{G}\right)=\sum_{i=0}^{n_{2}}\underset{0\leq j<n_{1}}{\min}\|p_{j}-q_{i}{\|}_{2}$$

Hence, the average distance (*ASSD*) is [Equation (4)]:

(4) $$ASSD=\frac{SD\left(S_{P},S_{G}\right)}{2n_{2}}+\frac{SD\left(S_{P},S_{G}\right)}{2n_{1}}$$

The Hausdorff distance (*HD*) is [Equation (5)]:

(5) $$HD=\max\left[SD\left(S_{P},S_{G}\right),SD\left(S_{P},S_{G}\right)\right]$$

In this experiment, the segmentation content includes four categories. Hence, when calculating the evaluation parameters, the label and prediction results are processed one-hot, and then the three evaluation parameters of each category are calculated according to the corresponding relationship.

### 4.4. Qualitative Evaluation of Three-Dimensional (3-D) Reconstruction

Using the trained neural network model, a section image (hereafter referred to as the section sequence) across all of the B-scan OCT images is segmented, and then the segmented section sequence is used for 3-D reconstruction. Here, the 3-D reconstruction of mouse skin is carried out by using the VTK module. For the three-dimensional image obtained from the 3-D reconstruction of the segmentation results, the hierarchical structure of mouse skin tissue and the edge contour of the damaged area can be clearly seen. This is of great significance for mastering the damage of skins. After the 3-D reconstruction, the segmented fault sequence is analyzed pixel by pixel, and the overall volume of the damaged area is calculated to realize a quantitative analysis of the damaged area, that is, the quantitative damaged volume of mouse skin induced by the laser. The formula for calculating the volume of the damaged area is [Equation (6)]:

(6) $$V=V_{voxel}\cdot \sum_{i=0}^{n}I\left(l_{i}\right)$$

where *V* represents the volume of the damaged area, *V_vixel_* represents the volume of each voxel, and the volume of a voxel in this experiment is *V_voxel_* = 10 μm × 22 µm × 50 μm = 11,000 μm^3^. *l_i_* corresponds to each slice sequence, and n represents the number of images contained in a slice sequence. In this experiment, n = 200. The *I*(*l_i_*) function judges the pixels in the segmentation result *l_i_* based on the judgment statement and obtains the number of voxels representing the damaged area by accumulation. To date, a combination of deep convolutional networks and quantitative calculations for analyzing the laser-induced damage of skins has been constructed.

## 5. Summary

In this study, an OCT image segmentation algorithm based on a deep neural network model was proposed for segmenting laser-induced skin damage, and the algorithms were compared and evaluated quantitatively for the segmentation of skin layers and laser-induced skin injuries. The results showed that the performance and effect based on the dual attention mechanism-based U-Net (attention and SE-based U-Net) model was the best. The Dice coefficient reached 0.90 in the dermis and injured area and 0.80 in the adipose layer and muscle layer. In the evaluation results, the ASSD and HD indicated that the segmentation results were good, with a high overlap rate and short edge distance with manually labeled regions. The method in this study achieved good results in the segmentation of OCT images of laser-induced skin lesions in mice and provides a good, intelligent quantitative analysis tool for the exploration of the biological effects of lasers. In the future, segmentation accuracy will be improved by optimizing the image processing and analysis algorithms, experimental data will be further visualized, and real-time intraoperative imaging of skin disease diagnosis and monitoring will be implemented. The results of this study have important application potential in future laser skin injury and repair and the diagnosis of skin disease.

## Figures and Tables

**Table 1:** Quantitative evaluation parameters of these deep network models for segmentation of OCT images.

Parameters	Models	DL	SFL	FML	DA
Dice	U-Net	0.92 ± 0.03	0.81 ± 0.11	**0.88 ± 0.04**	**0.91 ± 0.04**
	Atten-Unet	0.92 ± 0.03	0.84 ± 0.07	0.87 ± 0.05	0.88 ± 0.06
	SE-Unet	0.92 ± 0.04	0.84 ± 0.08	0.87 ± 0.05	0.90 ± 0.05
	SE-Atten-Unet	**0.93 ± 0.02**	**0.84 ± 0.08**	0.86 ± 0.06	**0.91 ± 0.04**
ASSD	U-Net	1.89 ± 1.23	**2.63 ± 0.91**	**1.28 ± 1.08**	7.19 ± 5.62
	Atten-Unet	2.16 ± 1.35	4.63 ± 3.56	1.31 ± 0.63	7.04 ± 4.16
	SE-Unet	2.02 ± 1.31	4.77 ± 3.86	1.29 ± 0.57	5.67 ± 3.76
	SE-Atten-Unet	**1.73 ± 0.74**	4.86 ± 4.02	1.39 ± 0.67	**5.07 ± 3.10**
HD	U-Net	25.06 ± 8.62	206.99 ± 30.76	19.73 ± 2.96	66.67 ± 43.25
	Atten-Unet	14.12 ± 10.13	31.54 ± 27.54	**4.70 ± 2.69**	29.16 ± 20.16
	SE-Unet	13.17 ± 10.35	**31.09 ± 29.09**	4.75 ± 2.75	22.36 ± 15.36
	SE-Atten-Unet	**9.87 ± 5.87**	31.27 ± 28.44	5.06 ± 3.09	**15.88 ± 7.88**

**Table 2:** Different radiation times and corresponding energy densities of supercontinuum laser.

Radiation Times (s)	1	2	3
Energy (J)	0.445	0.890	1.335
Radiation dose (J/cm^2^)	32.85	65.69	98.54

## Data Availability

The data that support the findings of this study are available from the corresponding author on reasonable request.

## References

[1] Huang D., Swanson E.A., Lin C.P., Schuman J.S., Stinson W.G., Chang W., Hee M.R., Flotte T., Gregory K., Puliafito C.A. (1991). Optical coherence tomography. Science.

[2] Hee M.R., Izatt J.A., Swanson E.A., Huang D., Schuman J.S., Lin C.P., Puliafito C.A., Fujimoto J.G. (1995). Optical coherence tomography of the human retina. Arch. Ophthalmol..

[3] Cobb M.J., Chen Y., Underwood R.A., Usui M.L., Olerud J., Li X. (2006). Noninvasive assessment of cutaneous wound healing using ultrahigh-resolution optical coherence tomography. J. Biomed. Opt..

[4] Atiyeh B.S., Gunn S.W., Hayek S.N. (2005). State of the art in burn treatment. World J. Surg..

[5] Srinivas S.M., de Boer J.F., Park H., Keikhanzadeh K., Huang H.E.L., Zhang J., Jung W.Q., Chen Z.P., Nelson J.S. (2004). Determination of burn depth by polarization-sensitive optical coherence tomography. J. Biomed. Opt..

[6] Park B.H., Saxer C., Srinivas S.M., Nelson J.S., de Boer J.F. (2001). In vivo burn depth determination by high-speedfiber-based polarization sensitive optical coherence tomography. J. Biomed. Opt..

[7] de Boer J.F., Srinivas S.M., Malekafzali A., Chen Z.P., Nelson J.S. (1998). Imaging thermally damaged tissue by polarization sensitive optical coherence tomography. Opt. Express.

[8] Pierce M.C., Sheridan R.L., Park B.H., Cense B., de Boer J.F. (2004). Collagen denaturation can be quantified in burned human skin using polarization-sensitive optical coherence tomography. Burns.

[9] Wan B., Ganier C., Du-Harpur X., Harun N., Watt F.M., Patalay R., Lynch M.D. (2021). “Applications and future directions for optical coherence tomography in dermatology. Br. J. Dermatol..

[10] Adan F., Nelemans P.J., Essers B.A.B., Brinkhuizen T., Dodemont S.R.P., Kessels J.P.H.M., Quaedvlieg P.J.F., Dermont G.J., Winnepenninckx V.J.L., Hamid M.A. (2022). Optical coherence tomography versus punch biopsy for diagnosis of basal cell carcinoma: A multicentre, randomised, non-inferiority trial. Lancet Oncol..

[11] Alawi S.A., Kuck M., Wahrlich C., Batz S., McKenzie G., Fluhr J.W., Lademann J., Ulrich M. (2013). Optical coherence tomography for presurgical margin assessment of non-melanoma skin cancer-a practical approach. Exp. Dermatol..

[12] Coleman A.J., Penney G.P., Richardson T.J., Guyot A., Choi M.J., Sheth N., Craythorne E., Robson A., Mallipeddi R. (2014). Automated registration of optical coherence tomography and dermoscopy in the assessment of sub-clinical spread in basal cell carcinoma. Comput. Aided. Surg..

[13] Iftimia N., Yélamos O., Chen C.-J., Maguluri G., Cordova M.A., Sahu A., Park J., Fox W., Alessi-Fox C., Rajadhyaksha M. (2017). Handheld optical coherence tomography-reflectance confocal microscopy probe for detection of basal cell carcinoma and delineation of margins. J. Biomed. Opt..

[14] de Carvalho N., Schuh S., Kindermann N., Kästle R., Holmes J., Welzel J. (2018). Optical coherence tomography for margin definition of basal cell carcinoma before micrographic surgery—Recommendations regarding the marking and scanning technique. Skin Res. Technol..

[15] Maier T., Cekovic D., Ruzicka T., Sattler E.C., Berking C. (2015). Treatment monitoring of topical ingenol mebutate in actinic keratoses with the combination of optical coherence tomography and reflectance confocal microscopy: A case series. Br. J. Dermatol..

[16] Malvehy J., Alarcon I., Montoya J., Rodríguez-Azeredo R., Puig S. (2016). Treatment monitoring of 0.5% 5-fluorouracil and 10% salicylic acid in clinical and subclinical actinic keratoses with the combination of optical coherence tomography and reflectance confocal microscopy. J. Eur. Acad. Dermatol. Venereol..

[17] Niculescu L., Bierhoff E., Hartmann D., Ruzicka T., Berking C., Braunmühl T.V. (2017). Optical coherence tomography imaging of basal cell carcinoma undergoing photodynamic therapy: A pilot study. Photodiagnosis Photodyn. Ther..

[18] Hussain A.A., Themstrup L., Nürnberg B.M., Jemec G. (2016). Adjunct use of optical coherence tomography increases the detection of recurrent basal cell carcinoma over clinical and dermoscopic examination alone. Photodiagnosis Photodyn. Ther..

[19] Markowitz O., Tongdee E., Levine A. (2019). Optimal cosmetic outcomes for basal cell carcinoma: A retrospective study of nonablative laser management. Cutis.

[20] Maier T., Kulichova D., Ruzicka T., Berking C. (2014). Noninvasive monitoring of basal cell carcinomas treated with systemic hedgehog inhibitors: Pseudocysts as a sign of tumor regression. J. Am. Acad. Dermatol..

[21] Boone M.A., Marneffe A., Suppa M., Miyamoto M., Alarcon I., Hofmann-Wellenhof R., Malvehy J., Pellacani G., del Marmol V. (2015). High-definition optical coherence tomography algorithm for the discrimination of actinic keratosis from normal skin and from squamous cell carcinoma. J. Eur. Acad. Dermatol. Venereol..

[22] Alex A., Weingast J., Hofer B., Eibl M., Binder M., Pehamberger H., Drexler W., Považay B. (2011). 3D optical coherence tomography for clinical diagnosis of nonmelanoma skin cancers. Imaging Med..

[23] di Ruffano L.F., Dinnes J., Deeks J.J., Chuchu N., Bayliss S.E., Davenport C., Takwoingi Y., Godfrey K., O’Sullivan C., Matin R.N. (2018). Cochrane Skin Cancer Diagnostic Test Accuracy Group. Optical coherence tomography for diagnosing skin cancer in adults. Cochrane Database Syst Rev..

[24] Boone M.A.L.M., Suppa M., Marneffe A., Miyamoto M., Jemec G.B., del Marmol V. (2016). A new algorithm for the discrimination of actinic keratosis from normal skin and squamous cell carcinoma based on in vivo analysis of optical properties by high-definition optical coherence tomography. J. Eur. Acad. Dermatol. Venereol..

[25] Lu J., Deegan A.J., Cheng Y., Liu T., Zheng Y., Mandell S.P., Wang R.K. (2021). Application of OCT-derived attenuation coefficient in acute burn-damaged skin. Lasers Surg. Med..

[26] Ahn Y., Lee C.Y., Baek S., Kim T., Kim P., Lee S., Min D., Lee H., Kim J., Jung W. (2016). Quantitative monitoring of laser-treated engineered skin using optical coherence tomography. Biomed. Opt. Express.

[27] Tognetti L., Cinotti E., Falcinelli F., Miracco C., Suppa M., Perrot J.-L., Rubegni P. (2022). Line-field confocal optical coherence tomography (LC-OCT) as a new tool for non-invasive differential diagnosis of pustular skin disorders. J. Eur. Acad. Dermatol. Venereol..

[28] Dubey K., Srivastava V., Dalal K. (2018). In vivo automated quantification of thermally damaged human tissue using polarization sensitive optical coherence tomography. Comput. Med. Imaging Graph..

[29] Fischman S., Pérez-Anker J., Tognetti L., di Naro A., Suppa M., Cinotti E., Viel T., Monnier J., Rubegni P., del Marmol V. (2022). Non-invasive scoring of cellular atypia in keratinocyte cancers in 3D LC-OCT images using Deep Learning. Sci. Rep..

[30] Ji Y., Yang S., Zhou K., Rocliffe H.R., Pellicoro A., Cash J.L., Wang R., Li C., Huang Z. (2022). Deep-learning approach for automated thickness measurement of epithelial tissue and scab using optical coherence tomography. J. Biomed. Opt..

[31] del Amor R., Morales S., Colomer A., Mogensen M., Jensen M., Israelsen N.M., Bang O., Naranjo V. (2020). Automatic Segmentation of epidermis and hair follicles in optical coherence tomography images of normal skin by convolutional neural networks. Front. Med..

[32] Pfister M., Schützenberger K., Pfeiffenberger U., Messner A., Chen Z., Santos V.A.d., Puchner S., Garhöfer G., Schmetterer L., Gröschl M. (2019). Automated segmentation of dermal fillers in OCT images of s mice using convolutional neural networks. Biomed. Opt. Express.

[33] Avanaki M., Hojjatoleslami A. (2013). Skin layer detection of optical coherence tomography images. Opt. -Int. J. Light Electron Opt..

[34] Kepp T., Droigk C., Casper M., Evers M., Hüttmann G., Salma N., Manstein D., Heinrich M.P., Handels H. (2019). Segmentation of mouse skin layers in optical coherence tomography image data using deep convolutional neural networks. Biomed. Opt. Express.

[35] Chou H.Y., Huang S.L., Tjiu J.W., Chen H.H. (2020). Dermal epidermal junction detection for full-field optical coherence tomography data of human skin by deep learning. Comput. Med. Imaging Graph..

[36] Fan Y., Ma Q., Xin S., Peng R., Kang H. (2021). “Quantitative and qualitative evaluation of supercontinuum laser-induced cutaneous thermal injuries and their repair with OCT images. Lasers Surg. Med..

[37] Fan Y., Ma Q., Liang J., Lu Y., Ni B., Luo Z., Cui Y., Kang H. (2019). Quantitative and qualitative evaluation of recovery process of a 1 064 nm laser on laser-induced skin injury: In vivo experimental research. Laser Phys. Lett..

[38] Ma Q., Fan Y., Luo Z., Cui Y., Kang H. (2021). Quantitative analysis of collagen and capillaries of 3.8-μm laser-induced cutaneous damage and wound healing. Lasers Med. Sci..

[39] Yu H., Gao J., Li A. (2016). Probability-based non-local means filter for speckle noise suppression in optical coherence tomography images. Opt. Lett..

[40] Fang L., Wang C., Li S., Rabbani H., Chen X., Liu Z. (2019). Attention to lesion: Lesion-aware convolutional neural network for retinal optical coherence tomography image classification. IEEE Trans. Med. Imaging.

[41] Hu J., Shen L., Albanie S., Sun G., Wu E. (2020). Squeeze-and-Excitation Networks. IEEE Trans. Pattern Anal. Mach. Intell..

